# Unlocking insights in real estate markets: Integrating geo-spatial data for comprehensive property valuation and urban planning

**DOI:** 10.1016/j.dib.2024.111141

**Published:** 2024-11-17

**Authors:** Serkan Varol, Serkan Catma

**Affiliations:** a Engineering Management and Technology Department, Serkan Varol, University of Tennessee at Chattanooga, TN 37403, USA; b Department of Business Administration, Serkan Catma, University of South Carolina Beaufort, Bluffton, SC 29910, USA

**Keywords:** Real estate markets, Housing data, Urban planning, Geo-spatial variables, Proximity to green recreational spaces, Air quality

## Abstract

This article summarizes a database for analyzing the impact of structural and environmental characteristics on residential property values in Hamilton County, TN. The original dataset consists of house characteristics data for 873 residential sales between January 1, 2023, and September 25, 2023. Using Googleʼs API tools and Point to Edge computations, several geographical variables—including the distance to green recreational areas, surrounding facilities, restaurants, air quality index, walk score—were gathered. The aggregated information can help forecast Hamilton County's housing market with accuracy and correctly assess the environmental impact on housing prices. More specifically, the value of any environmental amenity in the study area can be implicitly estimated using this dataset.

Specifications Table

**Table:** 

Subject	*Economics, Econometrics and Finance: Asset Pricing*
Specific subject area	*Geospatial data is included in house price assessments to create a more accurate and comprehensive picture of the complexities of real estate markets.*
Type of data	Type of data: xlsx-formatted Table, Figure, Graph Raw, Analyzed, Filtered, Processed
Data collection	*A tier I real estate company provided the dataset (House Characteristics), which includes 873 transactions of houses in Hamilton County, Tennessee, USA, between January 1, 2023, and September 25, 2023. Using the Alteryx end-to-end data analytics platform, redundant records were deleted, noisy data was eliminated, and data modification (such as attribute selection) was performed at the data preprocessing step. The distance-based variables (*e.g. *air quality index, driving distance to the closest restaurant) were retrieved using Google Cloud services. Furthermore, a Python script was created in order to obtain the walk score associated with each property. The distance between a set of point-type spatial objects and the closest edge of a group of line-type spatial objects was determined using Alteryx's point-to-edge computations.*
Data source location	*Location: Hamilton County, Tennessee, USA*
Data accessibility	***Please note:****All raw data referred to in this article must be made publicly available in a data repository prior to publication. Please indicate here where your data are hosted (the URL must be working at the time of submission and editors and reviewers must have anonymous access to the repository):* Repository name: Mendeley Data Data identification number: 10.17632/zp9cgjgngn.1 Direct URL to data: https://data.mendeley.com/datasets/zp9cgjgngn/1

## 1 Value of the Data

• The data includes information on various house characteristics, sales-specific information, factors based on distance, and dynamic variables depending on the environment.
• The data can be useful for analyzing the influence of green recreational spaces, which can be defined as lands consisting of playgrounds, trails, walking paths, and recreation centers, on property values. Spatial regression techniques can reveal potential economic benefits of adding new green recreational spaces so the city officials and planners can make informed decisions.
• The data can be utilized to assess the current real estate market and forecast property prices in the Chattanooga, Tennessee region. This is especially valuable for local governments who rely heavily on property taxes.
• The data can support the quantitative analysis in a way that the spatial aspect of this study can reveal important information about the neighborhoods with the least amount of access to parks and trails. The Hedonic pricing model can be implemented to assess the current distribution of green recreational spaces and identify the neighborhoods with accessibility issues and gentrification risks.
• Local and state officials can utilize the dataset to make decisions regarding the need for housing subsidies and other public policy programs to promote affordable housing. Academic investigators can have the opportunity to assess the relationship between property prices and various structural and environmental characteristics from a tempo-spatial perspective.

## 2 Background

The value of residential property is defined by a combination of factors that can be categorized as structural, locational, and neighborhood attributes. Neighborhood characteristics and proximity to environmental and other amenities need to be assessed in order to approximate the impact of each on property values. Therefore, the incorporation of geo-spatial data into home price analyses is an essential step to provide a more thorough and precise understanding of the complexities of real estate markets [1]. This enables stakeholders, including policymakers, sellers, buyers, and investors, to make more informed decisions. Specifically, the inclusion of location-specific factors, including but not limited to crime rates [2], air quality [3], proximity to green recreational spaces [4], restaurants [5], and grocery stores [5,6] can improve the market value of a property. However, gathering comprehensive and high-quality housing data that incorporates spatial aspects may pose challenges due to the potential for significant costs and time requirements [7,8]. The objective of this study is to provide a comprehensive dataset that builds a strong foundation for extracting significant insights into fields such as urban planning development and housing market analysis.

## 3 Data Description

The data publication (https://data.mendeley.com/datasets/zp9cgjgngn/1) consists of a single, xlsx-formatted dataset comprising forty columns. The data set comprises three primary components (1) house characteristics (highlighted in yellow) (2) environmental-related factors (highlighted in blue) (3) distance-based factors (highlighted in green). The variables for each component are identified and described in the subsequent section.

### 3.1 House characteristics

**Fig. 1:**
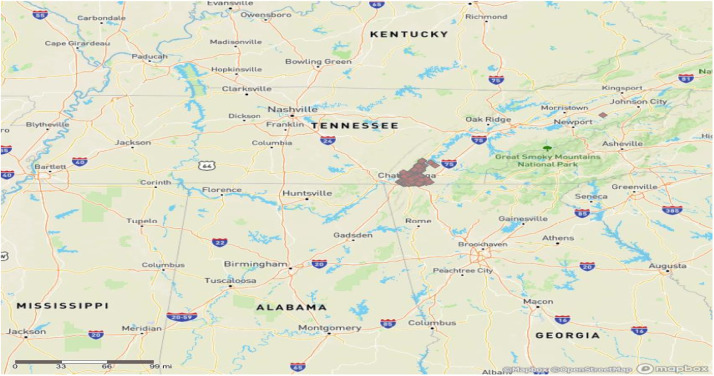
Home Sales from Jan 1, 2023 to September 25, 2023 in Hamilton Country, TN.

This original dataset which is acquired from a local real estate agency's MLS (Multiple Listing Service) listings comprises data pertaining to 873 residential (single family) sales that occurred in Hamilton County, Tennessee, between January 1, 2023, and September 25, 2023 (Fig. 1). The dataset includes information such as the listing price, number of bedrooms and bathrooms, square footage, and other similar details relevant to the housing market. The price of an average home sold in the study area is $411,708. 684 of the sold homes were financed with a conventional loan, 117 via Federal Housing Administration (FHA), 60 through Veteran Affairs (VA), 7 through the Tennessee Housing Development Agency, and 5 through other financing options (e.g., seller financing). Table 1 below contains home characteristics and their descriptions including but not limited to latitude and longitude information, building type, number of bedrooms, bathrooms, approximate square footage and acres, year built, seller concessions, transaction type, listing price, sold price, and sold terms.

**Table 1:** Property-specific variables and descriptions.

Variables	Description	Data Type/Sample
Longitude	Longitude of the property	Float / -77.XXXX
Latitude	Latitude of the property	Float / 38.XXXX
Zip Codes	5-digit zip code	String / “37,302”
Sold Month	The month that the property was sold	String / “January”
Days on Market	The day difference from listing to under contract	Double / 50
Orig. List Price	Original list price of the property	Double / $300,123
Listing Price	Listing price by the date of the contract	Double / $560,020
Sold Price	The sold price of the property	Double / $250,000
Taxes	Paid property tax	Double / $2554
Sold Term	Transaction type	String / “Conventional Loan”
Sqft-Total	Total square footage of the property	Double / 2200
List Price/Sqft	Listing price per sqft	Double / $223
Sold Price/Sqft	Actual sold price per sqft	Double / $226
Basement	Basement availability	Boolean / Yes-No
Year Built	The built year of the house	String / “2024”
Aprx.Acres	Approx size of the land	Double / 0.08
Total Bedroom	Number of bedrooms	Double / 5
Total Bathrooms	Number of bathrooms	Double / 2
HOA	Homeowners’ association availability	Boolean / Yes-No
Warranty	Home sold with warranty or not	Boolean / Yes-No
Seller Concession	Costs that the seller agrees to pay	Boolean / Yes-No
New Construction	Whether the property is new or used	Boolean / Yes-No

### 3.2 Neighborhood characteristics

Environmental factors have a substantial impact on the determination of home sale prices, reflecting a location's perceived risks as well as its overall quality of life [9]. In this study, we have investigated three key subjects: (1) crime rate data, (2) school ratings, and (3) air quality ratings, all of which have been discovered to have an effect on willingness to purchase a house. Table 2 contains an explanation of variables on each subject.

1. *Crime rate data*: Crimegrade.org, a website that compiles data from all available police departments and federal agencies based on postal codes, provides crime rate information (overall crime rate, violent crime rate, property crime rate, and other crime rate) for each zip code. It calculates crime rates by using data primarily from the FBI's Uniform Crime Reporting (UCR) program and local law enforcement reports. Crime figures are normalized based on population size to articulate crime rates as the number of crimes per 1000 residents. The overall crime rate includes fields such as violent crimes (e.g., murder, assault, robbery), property crimes (e.g., theft, burglary, arson), and other crimes (e.g., vandalism, identity theft, kidnapping, animal cruelty). Crime rates are measured on a scale ranging from *A*+ to F, with *A*+ indicating a significantly lower than average crime rate in the United States, and F indicating a significantly higher rate [10]. Fig. 2 depicts the overall crime rate of the study area.

**Fig. 2:**
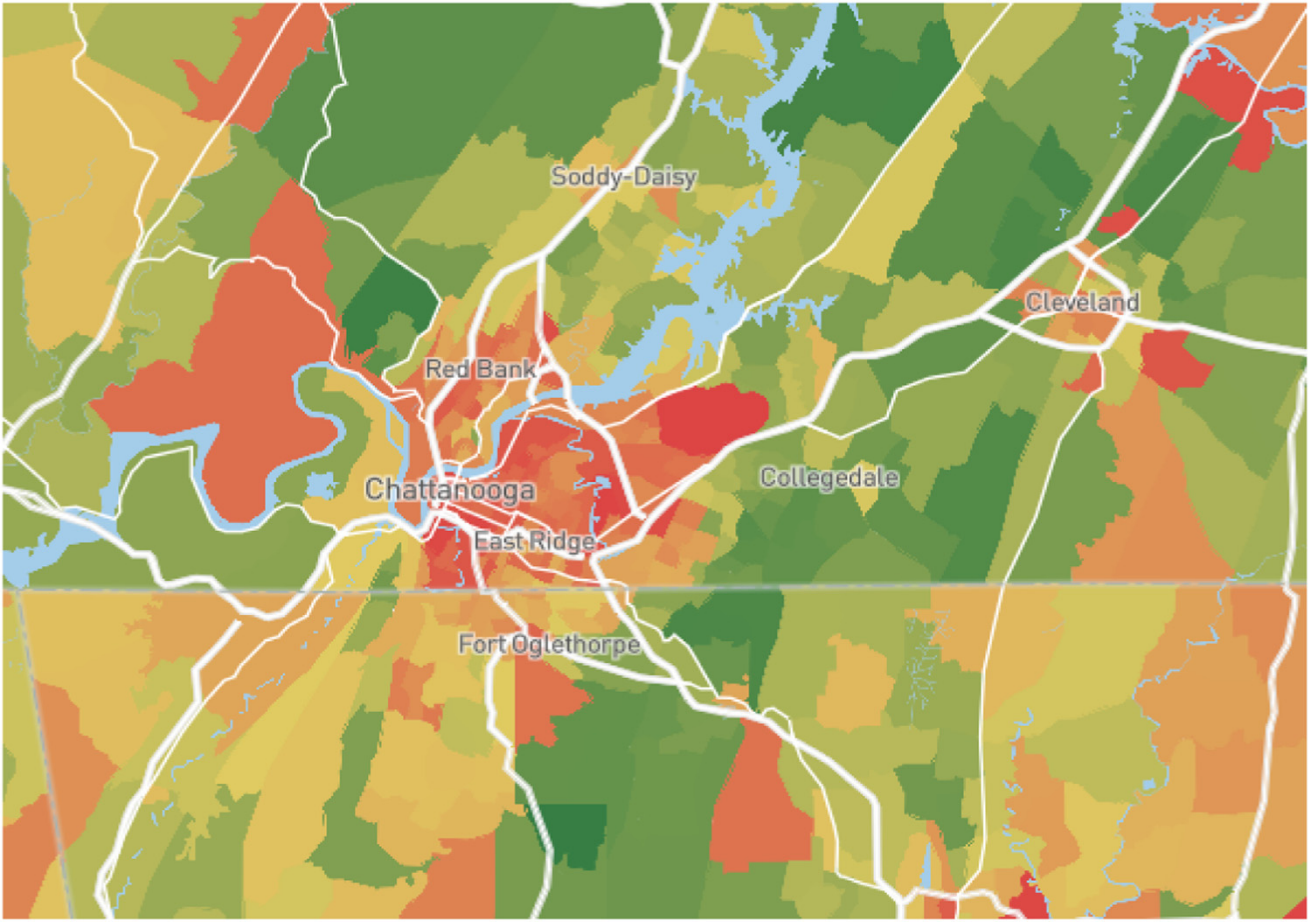
Overall crime rate of Hamilton county.
2. *School ratings*: The data on school ratings is extracted from the website greatschools.org (a nonprofit organization). The overall rating computation (ratings follow a 1–10 scale, where 10 is the highest and 1 is the lowest) relies on four distinct ratings, namely the student progress rating or academic progress rating, college readiness rating, equity rating, and test score rating, each intended to assess various aspects of school achievement [11].
3. *Air quality data*: The air quality data was obtained from Google Maps platforms. Google employs an air quality model that utilizes a multi-layered methodology. The utilized methodology integrates data from NO, NOx, and Non-Methane Hydrocarbons and other several input sources (e.g., governmental reference monitoring station, satellite information, meteorology, and commercial sensor networks), then scientifically evaluates the layers. The API provides the values of different air quality indexes with a precision of 500 × 500 m for each latitude and longitude [12].
4. *Walk Score*: The walk score is sourced from walkscore.com. It is a metric used to assess a placeʼs walkability based on population density, pedestrian infrastructure, and the distance to amenities. A proprietary algorithm is utilized to produce a score of 0 to 100 for a specific location, with higher values indicating greater walkability [13].

**Table 2:** Environmental factors and descriptions.

Variables	Description	Data Type/Sample
Overall Crime Rate	The average rating for violent, property, and other crimes	String / “*A*+”
Violent Crime Rate	Assault, robbery, rape, and murder rating per 1000	String / “F”
Property Crime	Theft, Vehicle theft, burglary, arson rating per 1000	String / “*B*+”
Other Crime	Kidnapping, vandalism, identity theft, cruelty per 1000	String / “*A*+”
Elementary School	Name of the elementary school for the property	String / “Apison”
EM-Rating	Elementary school Rating	Double-Ordinal / 7
Middle School	Name of the middle school for the property	String / “Tyner”
MS-Rating	Middle school rating	Double-Ordinal / 5
High School	Name of the high school for the property	String / “Ooltewah”
HS-Rating	Highschool rating	Double-Ordinal / 6
Air Quality Index	Air quality indexes with each latitude and longitude	Double / 67
Walk Score	Walkability score for each property address	Double / 91

### 3.3 Distance based factors

The influence of distance-based considerations on property values is significant. The variables considered in this study compromise the (1) characteristics of the surrounding green recreational spaces, (2) the number of restaurants nearby, and (3) the distance to the closest grocery store, which have been defined in Table 3.

1. *Characteristics of green recreational spaces*: Green recreational spaces can have an advantageous effect (favorable impact on the value of real estate) on property values by increasing the appeal, livability, and attractiveness of areas [4]. However, the exact effect may differ depending on factors such as the type and size of the green areas and the accessibility of those locations [14]. In this study, the point-to-point distance (in miles) between each property and the (a) closest playground, (b) trail, (c) walking path, and (d) recreation center was measured.
2. *Number of restaurants nearby*: In this study, the number of restaurants (e.g., dine-in, fast food) within a 15-minute driving distance was obtained. Google API limits the maximum quantity at 20 (there were locations where the number of restaurants was marked as “20+”), thus the data has been translated into categorical variables: level 5 indicates 20+ restaurants, level 4 16–19, level 3 11–15, level 2 6–10, and level 1 1–5. The decision to categorize the number of nearby restaurants was made due to the limitations imposed by the Google API. By grouping the data into categorical variables, we ensured a more consistent comparison across observations. However, this transformation may potentially reduce the precision of the analysis because it introduces broader categories instead of using exact counts.
3. *Distance to the nearest grocery shop*: The driving distance (in miles) to the nearest grocery store is calculated for each location based on latitude and longitude information.

**Table 3:** Distance based factors and descriptions.

Variables	Description	Data Type/Sample
Trial Hike Dist	Point to edge distance to the nearest trial-hike	Double / 5.2 (miles)
Walking Path Dist	Point to edge distance to the nearest walking path	Double / 3.0 (miles)
Playground Dist	Point to edge distance to the nearest playground	Double / 2.9 (miles)
RecCenter Dist	Point to edge distance to the nearest recreational center	Double / 0.8 miles
Rest Nearby	Number of restaurants within a 15-min driving distance	String / “Level 5”
Dist to Grocery	Driving distance to the nearest grocery shop	Double / 3.8 (miles)

## 4 Experimental Design, Materials and Methods

**Fig. 3:**
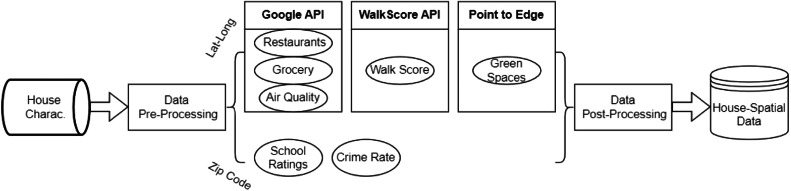
Taxonomy of the dataset.

The structural arrangement and methodological framework utilized in the compilation of the data are illustrated in Fig. 3. The visualization depicts the sequential process of collecting and integrating data from various sources, the stages involved in data processing and retrieving environmental-related and distance-based factors through approaches such as application programming interface (API) platforms and point-to-edge calculations.

The dataset (House Characteristics) comprising 873 sales of residential houses in Hamilton County, Tennessee, from January 1, 2023, to September 25, 2023, is sourced from a tier I real estate provider. This dataset includes a variety of features, such as property characteristics, amenities, and the latitude and longitude information of the properties. This dataset was pre-filtered by the real estate provider to exclude all commercial sales (e.g., business buildings). . During the data preprocessing stage, redundant records were removed (e.g., the houses outside Hamilton County were removed), noisy data was eliminated (e.g., the dataset includes only single-family houses, excluding mansions and estates), and data modification (such as attribute selection) was carried out (e.g., certain attributes such as agency names, under-contract date, full address), using the Alteryx end-to-end data analytics platform.

### 4.1 API services

Initially, we set up google cloud and enabled places API. Then, we obtained an API key from the Google Cloud Console to authenticate a request to the Google Cloud API (Directions API, Distance Matrix API, Places API, and Air Quality API services). A Python script was used to generate a request (via Places API) for nearby restaurants within a 100-mile radius. After obtaining a compilation of restaurants in close proximity, the Directions API was employed to compute driving directions from each property to the restaurants and locate the number of restaurants within 15 min of driving distance. Using the same approach, (the Places API and Directions API were utilized), a Python script was also developed to calculate the driving distance between each property and the closest grocery store.

Air quality (AQ) data was acquired through the utilization of the Air Quality API, which presents the values of air quality indices with a 500 × 500-m accuracy for each latitude and longitude. Although the output of AQ API offers several information, such as recommendations for reducing the effects of air pollutants and providing tailored guidance for specific populations, these data fields have not been included in the retrieved data as they were not relevant. However, air pollution data could be highly relevant for other studies, particularly those focused on public health and urban planning. Likewise, the walk score was acquired by providing longitude and latitude data for each property through the API of the Walk Score website (Walkscore.com). Walk Score evaluates walkability based on several key factors, including the proximity to amenities such as grocery stores, schools, parks, and restaurants. It also considers population density, as denser areas typically have more accessible services. Additionally, the score reflects pedestrian friendliness by assessing street connectivity, the presence of sidewalks, and safety features for walkers. Together, these factors help provide a comprehensive measure of how walkable a specific location is. We provide the Python script/code used to generate our API calls [15]

### 4.2 Point-to-Edge calculations

**Fig. 4:**
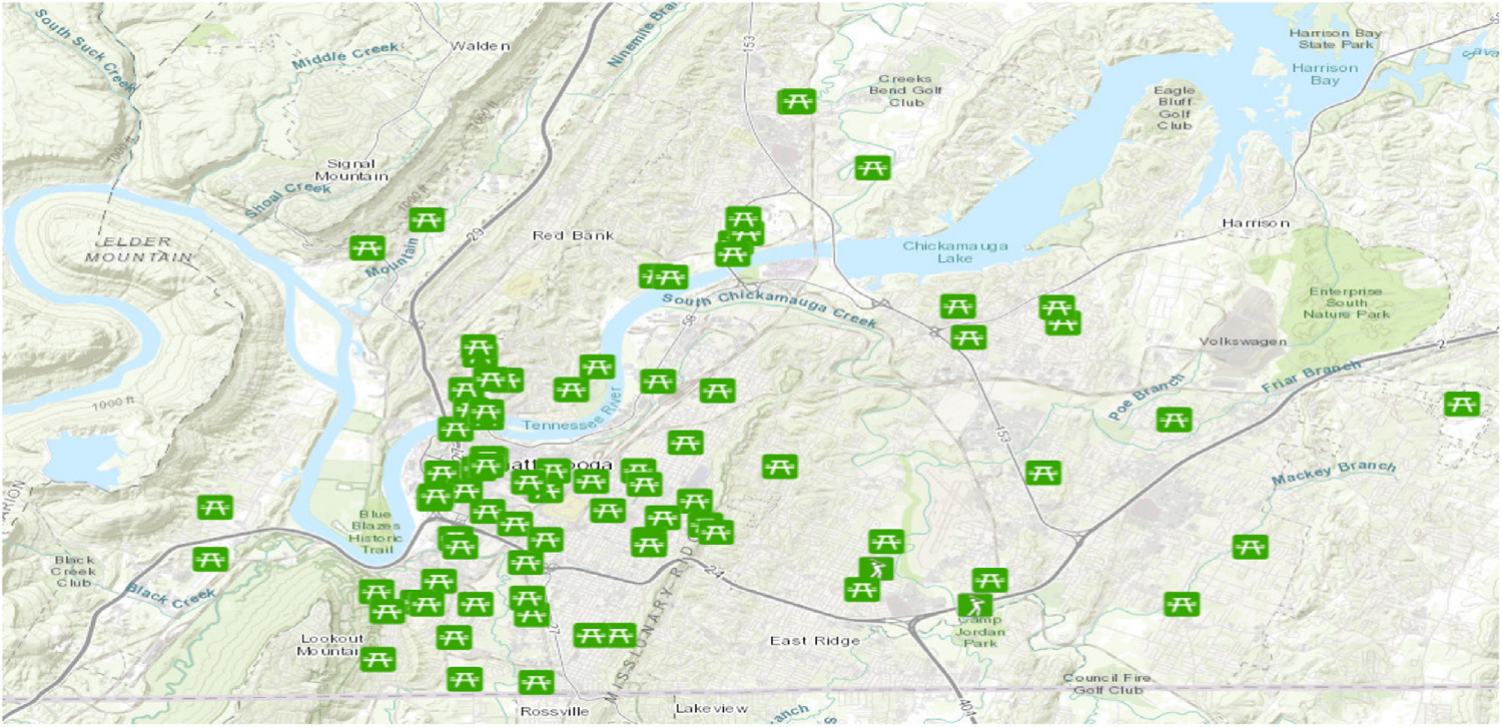
Green recreational spaces in Hamilton county.

**Fig. 5:**
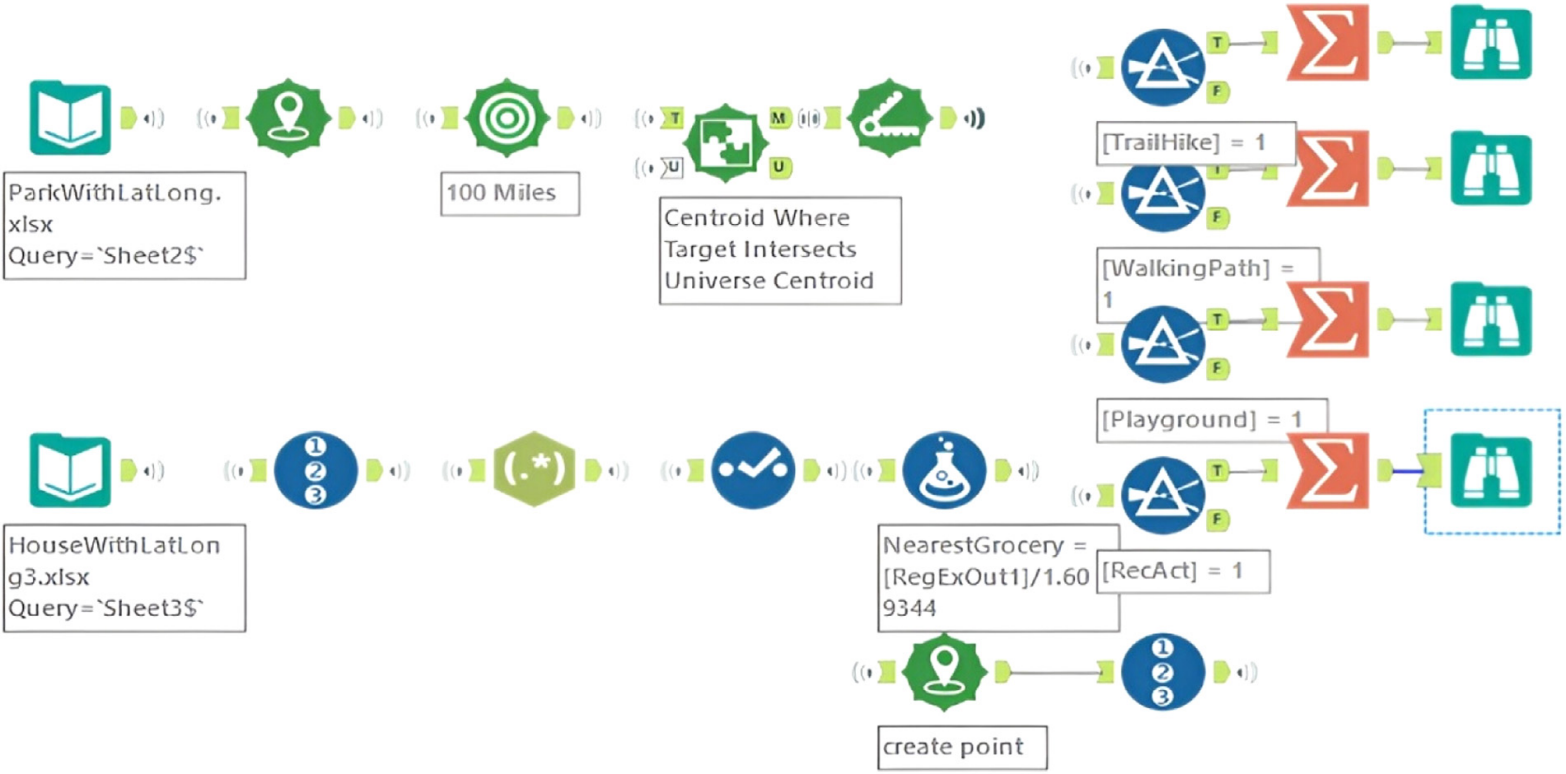
Alteryx geocoder setup.

The Chattanooga Department of Parks and Outdoors provides a comprehensive list of green recreational spaces (Fig. 4), including community and neighborhood parks, greenways, and trails, along with their respective full addresses [16]. We used the geocoder macro (planer distance is used) on the Alteryx platform (Fig. 5) to retrieve the longitude and latitude points from these addresses. Then, the distance tool (point to edge-line distance) was utilized to determine the distance between each property and the nearest green areas. This tool was also utilized to calculate the distance between a set of point-type spatial objects and the nearest edge of a collection of line-type spatial objects.

It is important to note that errors in point-to-edge distance calculations and geocoding may occur due to (1) spatial data quality (e.g., outdated spatial datasets may potentially alter points and edges this introduces errors), (2) geocoding precision (e.g., outdated addresses), (3) other factors such as misalignment in coordinate systems and preferred method (planar and spherical distance) for distance calculations. Errors can vary from 10 to 50 m for geocoding in urban areas.

### 4.3 Zip code-based variables

Variables such as crime rates and school rating scores were derived from zip codes (there are 26 available in the original data set) due to the lack of these statistics through latitude and longitude. The Crime rate data is sourced from crimegrade.org. Similarly, the data on school ratings is extracted from the website greatschools.org using properties’ zip codes. Using zip code-level data for crime rates and school ratings has limitations due to its lack of specificity and arbitrary boundaries. It may potentially mask significant local variances. For instance, variations within different neighborhoods may be overlooked when the data is averaged across the entire zip code. Therefore, even while certain areas may be significantly safer or have better schools than others, the statistics may indicate that a zip code across the entire area has a particular level of crime or school quality. Using address level or point-based data (If discovered) can provide better insights into where issues are present.

## Limitations

The house-spatial dataset presents some limitations. First, the comprehensiveness of crime rate data may be limited. Frequently, crime data comprise confidential details related to individuals and occurrences. Further legal and ethical considerations may arise concerning the disclosure and distribution of this information. While CrimeGrade.org asserts that they get extensive data from all available police departments, they also acknowledge that numerous police departments lack transparency in their reporting. To address these substantial data deficiencies, they utilized machine learning techniques. Therefore, obtaining precise crime data can be challenging. Another limitation of this study is the dynamic nature of air quality, which is susceptible to frequent changes that result from a variety of factors related to weather, human activities, and geographical features. Such variability in air quality has the potential to impact the accuracy and dependability of the gathered and examined data. As a result, although our results offer helpful insights into the prevalent air quality circumstances throughout the study, they might not comprehensively represent all temporal fluctuations.

## Ethics Statement

The authors have read and followed the ethical requirements for publication in Data in Brief and confirm that the current work does not involve human subjects, animal experiments, or any data collected from social media platforms.

## CRediT Author Statement

**Serkan Varol:** Data curation, Methodology, Investigation, Formal Analysis. **Serkan Catma**: Conceptualization, Validation, Original draft preparation, Writing- Reviewing and Editing.

## Data Availability

Mendeley DataThe Geospatial Housing Dataset for Hamilton County (Original data).
